# Coherent phonon source based on electron spin resonance in a quantum-dot qubit

**DOI:** 10.1038/s41598-025-96345-4

**Published:** 2025-04-19

**Authors:** J. Fransson, J. P. Bird

**Affiliations:** 1 Department of Physics and Astronomy, Uppsala University, Box 516, 75120 Uppsala, Sweden; 2 Department of Electrical Engineering, University at Buffalo, The State University of New York, Buffalo, NY 14260 USA

**Keywords:** Nanoscale devices, Physics, Condensed-matter physics

## Abstract

One of the key requirements for quantum phononics, especially for scenarios involving quantum communication and quantum-state transduction, is the implementation of a controlled *phonon source*, ultimately with the capacity to source single phonons at some desired generation rate. In this article, we describe a scheme for the controlled sourcing of phonons that exploits an electron-spin resonance between the Zeeman-split levels of a gated quantum dot. This on-chip scheme allows for broad tunability of the energy of the generated phonons, with convenient electrical control. By providing a compact and coherent source, this scheme is also well suited to the construction of more-extended phononic circuits, involving the sourcing, transmission and detection of phononic signals.

## Introduction

Quantum phononics^1^ is a field which seeks to overturn the traditionally accepted viewpoint that phonons are a problematic source of decoherence in quantum systems, seeking instead to harness these elementary excitations of the crystal lattice. In traditional treatments of these bosonic quanta, they are typically viewed as serving as an effective thermalizing reservoir, whose coupling to electronic or to other excitations is able to very effectively erase quantum coherence (or memory). Recently, however, it has come to be appreciated that, when excited selectively in sufficiently small numbers, phonons can serve as an effective means to mediate the long-range coupling between quantum bits (qubits)^2^. As one paradigm for this approach, substantial experimental and theoretical progress has been made in utilizing mechanical resonators to prepare specific acoustic modes and to transduce between electrical, optical and mechanical quanta^3–7^. Phononic coupling is also of interest as a means to couple vacany-center defined qubits, and to engineer the quantum coherence of such elements^2^.

One of the key requirements for quantum phononics, especially for scenarios involving quantum communication and quantum state transduction, is the implementation of a controlled *phonon source*. In many situations, it is desirable to be able to achieve such sourcing at the single-phonon level, at some desired generation rate. A variety of different approaches to this problem have been proposed, including schemes that rely on photonic coupling to vacancy centers or to optomechanical circuits^8,9^, as well as coherent phonon generation in twisted bilayer graphene^10^. Experimental demonstrations of such sources have also recently been reported, including approaches that rely upon the optical excitation of phonons in bulk crystals and in mechanical resonators^8,9,11,12^.

**Fig. 1:**
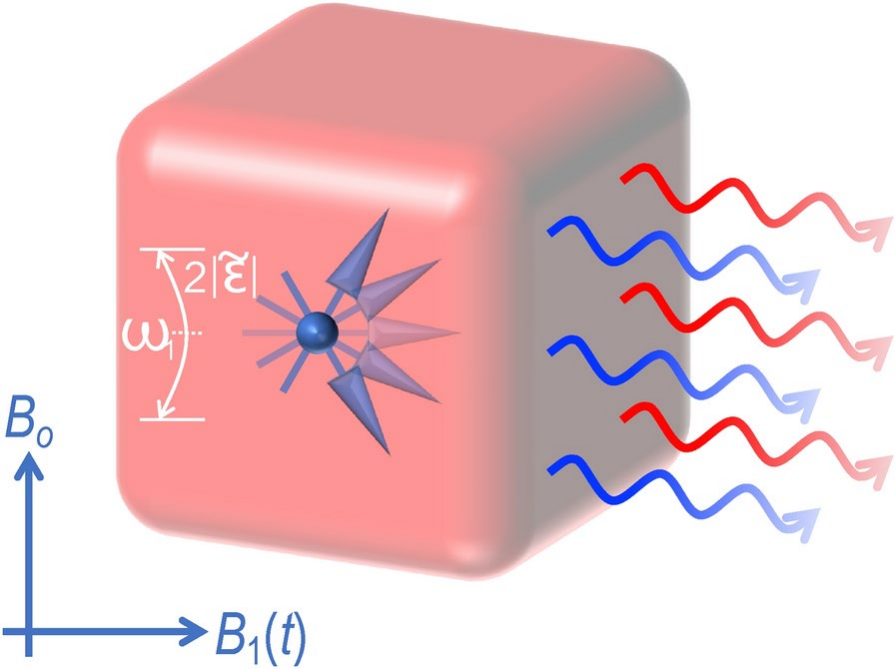
Schematics of the energy setting. (**a**) The static magnetic field $\textbf{B}_0=B_0\hat{\textbf{z}}$ induces a Zeeman splitting $2|\tilde{\varvec{\varepsilon }}_1|$ of an electronic level in a QD (denoted schematically by the dotted line), while the time-dependent magnetic field $\textbf{B}_1(t)$ drives excitations between these two spin states. (**b**) This process in turn generates phonons at energies $\omega _1\pm 2|\tilde{\varvec{\varepsilon }}_1|$.

For many potential applications of quantum phononics, electrically rather than optically driven sourcing of phonons is desired, as it provides a natural means to realize chip-based control of phonons. In this work we therefore describe a scheme for the controlled sourcing of phonons. These sources exploit the discrete level structure of electronic QDs, which can be defined in relatively straightforward fashion by means of electrostatic gating^13^. Phonon sourcing is achieved by exploiting an electron-spin resonance (ESR) between Zeeman-split levels (see Fig. 1), a scheme that allows for broad tunability of the energy of the generated phonons. Since the microwave field required for the resonance can be generated from an on-chip microstrip line^14^, while the bias field can be supplied with permanent nanomagnets, our approach is an all-electrical one that should be well suited to fully on-chip applications, without the need for optical coupling.

## Results and discussion

The physical system that we consider is an electrostatically defined (gated) quantum dot (QD)^13^, indicated schematically in Fig. 1. Such structures have previously been widely used to implement prototypical spin-based qubits^15^, with dynamical readout^16^ and microwave ESR^14^ both having been demonstrated. To describe the phonon sourcing function of this system, we decompose its Hamiltonian as ${{\mathcal {H}}}={{\mathcal {H}}}_\text {QD}+{{\mathcal {H}}}_\text {ph}+{{\mathcal {H}}}_\text {e-p}$, where ${{\mathcal {H}}}_\text {QD}$ is the Hamiltonian of the isolated QD, ${{\mathcal {H}}}_\text {ph}$ describes the phonons of the host material, and ${{\mathcal {H}}}_\text {e-p}$ represents the influence of the coupling of the electronic levels of the dot to the phonons. The terms ${{\mathcal {H}}}_\text {QD}$ and ${{\mathcal {H}}}_\text {e-p}$ are expressed as

1a $$\begin{aligned} {{\mathcal {H}}}_\text {QD}=&\psi ^\dagger {\varvec{\varepsilon }}\psi +\psi ^\dagger \textbf{B}_1(t)\psi , \end{aligned}$$

1b $$\begin{aligned} {{\mathcal {H}}}_\text {e-p}=&\sum _\textbf{q}\psi ^\dagger {\varvec{\Lambda }}_\textbf{q}\psi \Bigl (b_\textbf{q}+b^\dagger _{{\bar{\textbf{q}}}}\Bigr ) . \end{aligned}$$

A brief derivation of the model is outlined in a separate section below. First, however, we introduce the physical content accounted for by this model.

In Eq. (1a), the $2\times 2$-matrix ${\varvec{\varepsilon }}=\varepsilon _{0}\sigma ^0+g\mu _BB_0\sigma ^z/2$ defines the energy spectrum of the QD under a static external magnetic field $B_0$, the spinors $\psi ^\dagger =(\psi _\uparrow ^\dagger \ \psi _\downarrow ^\dagger )$ and $\psi =(\psi _\uparrow \ \psi _\downarrow )^t$ define creation and annihilation, respectively, of electrons in the QD at energy $\varepsilon _{0}$ (where *g* and $\mu _B$ are the gyromagnetic ratio and Bohr magneton, respectively). A time-dependent external magnetic field $\textbf{B}_1(t)=g\mu _BB_1(e^{-i\omega _1t}\sigma ^++H.c.)/2$ is applied to generate spin-flip transitions in the conventional ESR configuration.

The Hamiltonian in Eq. (1b) describes the electron-phonon coupling as ${\varvec{\Lambda }}_\textbf{q}=\lambda _\textbf{q}\sigma ^0+{\varvec{\lambda }}_\textbf{q}\cdot {\varvec{\sigma }}$, where the scalar coupling $\lambda _\textbf{q}$ defines the spin-independent coupling whereas the vector ${\varvec{\lambda }}_\textbf{q}=(\lambda _\textbf{q}^{(x)},\lambda _\textbf{q}^{(y)},\lambda _\textbf{q}^{(z)})$ provides a vibrationally induced spin-orbit coupling.^17,18^ Furthermore, $b_\textbf{q}+b^\dagger _{{\bar{\textbf{q}}}}$ represents the nuclear displacement in terms of phonon quantum operators at the momentum/wave vector $\textbf{q}$ (${\bar{\textbf{q}}}=-\textbf{q}$), associated with the energy of the phonon reservoir ${{\mathcal {H}}}_\text {ph}=\sum _\textbf{q}\omega _\textbf{q}b^\dagger _\textbf{q}b_\textbf{q}$. Finally, $\sigma ^0$ and ${\varvec{\sigma }}=(\sigma ^x,\sigma ^y,\sigma ^z)$ denote the unit matrix and vector of the Pauli matrices.

Application of the unitary transformation $e^{\mathcal {S}}{{\mathcal {H}}}e^{-{\mathcal {S}}}+i({\partial _t}e^{\mathcal {S}})e^{-{\mathcal {S}}}=\tilde{{\mathcal {H}}}$ (where ${\mathcal {S}}=i\omega _1ts^z$ and $s^z=\psi ^\dagger \sigma ^z\psi /2)$, leads to a time-independent form of $\tilde{{\mathcal {H}}}_\text {QD}$, with $\tilde{\varvec{\varepsilon }}={\varvec{\varepsilon }}-\omega _1\sigma ^z/2$. Simultaneously, the electron-phonon coupling Hamiltonian is modified to

2 $$\begin{aligned} \tilde{{\mathcal {H}}}_\text {e-p}=&\sum _\textbf{q}\psi ^\dagger \tilde{\varvec{\Lambda }}_\textbf{q}(t)\psi \Bigl ( b_\textbf{q}+b_{-\textbf{q}}^\dagger \Bigr ) . \end{aligned}$$

In this rotating reference frame, the time-dependent coupling between the QD and the phonon reservoir is given by

3 $$\begin{aligned} \tilde{\varvec{\Lambda }}_\textbf{q}(t)=&\lambda _\textbf{q}\sigma ^0 + \begin{pmatrix} \lambda _\textbf{q}^{(x)}\cos \omega _1t-i\lambda _\textbf{q}^{(y)}\sin \omega _1t \\ \lambda _\textbf{q}^{(y)}\cos \omega _1t+i\lambda _\textbf{q}^{(x)}\sin \omega _1t \\ \lambda _\textbf{q}^{(z)} \end{pmatrix} \cdot {\varvec{\sigma }}. \end{aligned}$$

As we shall see below, this transfer of the time-dependence into the electron-phonon coupling opens up an interpretation of the coupling parameters $\tilde{\varvec{\lambda }}_\textbf{q}(t)$ that is analogous to classical spin variables. We shall, henceforth, assume that $\lambda _\textbf{q}^{(z)}=0$, for all *q*, since there is no time-dependence connected to this component.

### Derivation of the model

The model for our investigation can be derived in the following way. Consider a localized spin embedded in an electronic band structure which is coupled to phonons. A model for such a set-up is given by

4 $$\begin{aligned} {{\mathcal {H}}}=&\sum _\textbf{k}\psi ^\dagger _\textbf{k}{\varvec{\varepsilon }}_\textbf{k}\psi _\textbf{k}+ \psi ^\dagger {\varvec{\varepsilon }}\psi + \sum _\textbf{k}\Bigl ( u_\textbf{k}\rho _\textbf{k}\psi ^\dagger \psi + v_\textbf{k}\textbf{s}_\textbf{k}\cdot \textbf{s}\Bigr ) + \sum _{\textbf{k}\textbf{q}} M_{\textbf{k}\textbf{q}}\rho _\textbf{k}\Bigl (b_\textbf{q}+b^\dagger _{{\bar{\textbf{q}}}}\Bigr ) , \end{aligned}$$

where $\psi _\textbf{k}$ is the spinor for the band electrons with wave vector $\textbf{k}$ and energy defined energy matrix ${\varvec{\varepsilon }}_\textbf{k}$, ${\varvec{\varepsilon }}$ defines the energy of the localized spin $\textbf{s}=\psi ^\dagger {\varvec{\sigma }}\psi /2$, and the quantities $\rho _\textbf{k}=\sum _{{\varvec{\kappa }}}\psi ^\dagger _{\textbf{k}+{\varvec{\kappa }}}\psi _{{\varvec{\kappa }}}$ and $\textbf{s}_\textbf{k}=\sum _{{\varvec{\kappa }}}\psi ^\dagger _{\textbf{k}+{\varvec{\kappa }}}{\varvec{\sigma }}\psi _{{\varvec{\kappa }}}/2$ define the charge density coupled to the phonons with rate $M_{\textbf{k}\textbf{q}}$ and spin density coupled to the localized spin with rate $v_\textbf{k}$, respectively.

An effective model for the localized spin coupled to the phonons can be obtained by integrating out the electrons in the band structure, using the formalism developed in Ref. 19. This procedure leads to the effective Hamiltonian

5 $$\begin{aligned} {{\mathcal {H}}}_\text {e-p}=&\sum _\textbf{q}\Bigl ( \psi ^\dagger \psi \lambda _\textbf{q}+ 2\textbf{s}\cdot {\varvec{\lambda }}_\textbf{q}\Bigr ) \Bigl (b_\textbf{q}+b^\dagger _{{\bar{\textbf{q}}}}\Bigr ) = \sum _\textbf{q}\psi ^\dagger \Bigl ( \lambda _\textbf{q}\sigma ^0 + {\varvec{\lambda }}_\textbf{q}\cdot {\varvec{\sigma }}\Bigr ) \psi \Bigl (b_\textbf{q}+b^\dagger _{{\bar{\textbf{q}}}}\Bigr ) , \end{aligned}$$

for the coupling between the localized spin and the phonons. It should be remarked that the procedure also yields indirect spin-spin, spin-charge, charge-charge, and phonon-phonon interactions that have either no influence or do not qualitatively change the physics of the coupling between the spin and phonons. These contributions are, therefore, omitted in the following.

In this effective model, the interactions $\lambda _\textbf{q}=\sum _{\textbf{k}\textbf{k}'}u_\textbf{k}\langle {\mathop {\langle \rho _\textbf{k}|\rho _{\textbf{k}'}\rangle }}\rangle M_{\textbf{k}'\textbf{q}}$ and ${\varvec{\lambda }}_\textbf{q}=\sum _{\textbf{k}\textbf{k}'}v_\textbf{k}\langle {\mathop {\langle \textbf{s}_\textbf{k}|\rho _{\textbf{k}'}\rangle }}\rangle M_{\textbf{k}'\textbf{q}}$ are mediated by the underlying electronic structure. The structure of the propagators $\langle {\mathop {\langle \rho _\textbf{k}|\rho _{\textbf{k}'}\rangle }}\rangle$ and $\langle {\mathop {\langle \textbf{s}_\textbf{k}|\rho _{\textbf{k}'}\rangle }}\rangle$ demonstrates that the former is of purely charge nature whereas the latter also depends on the spin in the electronic structure. In Ref. 19 it was demonstrated that the spin-phonon interaction is non-vanishing if the electronic structure sustains either a spin-density and/or spin-currents, where the latter implies the existence of an effective spin-orbit coupling. Either condition can be parametrized in terms of the electronic spectrum, such that ${\varvec{\varepsilon }}_\textbf{k}=\varepsilon _{\textbf{k}}^{(0)}\sigma ^0+{\varvec{\varepsilon }}_\textbf{k}^{(1)}\cdot {\varvec{\sigma }}$, where the scalar $\varepsilon _{\textbf{k}}^{(0)}$ and vector ${\varvec{\varepsilon }}_\textbf{k}^{(1)}$ account for the spin-independent and spin-dependent contributions, respectively, to the electronic energies. In terms of these quantities, the spin-phonon coupling exists whenever ${\varvec{\varepsilon }}_\textbf{k}^{(1)}\ne 0$. Hence, an underlying assumption in this investigation is that the material the localized spin is embedded in has a non-zero spin-orbit coupling. This can have various origins, including bulk inversion asymmetry, leading to Dresselhaus type of spin-orbit coupling, or Rashba type spin-orbit coupling at a surface or heterostructure interface.^20^

### Phonon excitations

The first scenario we consider is that of continuous phonon pumping at a specific frequency ($\omega _1$) of interest. This problem reduces to one of determining the phonon occupation $\langle {n_\textbf{q}}\rangle (t)=\langle {b^\dagger _\textbf{q}b_\textbf{q}}\rangle (t)$ as a function of the time-dependent modulation of the system. Since the lesser phonon Green function $D_{\textbf{q}\textbf{q}'}^<(t,t')$ ($=(-i)\langle {Q_{{\bar{\textbf{q}}}'}(t')Q_\textbf{q}(t)}\rangle$, where $Q_\textbf{q}=b_\textbf{q}+b_{{\bar{\textbf{q}}}}^\dagger$) is proportional to the density of occupied phonon states, it can be used to study the phonon excitations. However, the occupation number $\langle {n_\textbf{q}}\rangle (t)$ and $D_{\textbf{q}\textbf{q}}^<(t,t)$ are not identical. Rather, these two quantities are connected via the identity^21^

6 $$\begin{aligned} \langle {n_\textbf{q}}\rangle (t)=&\lim _{{\mathop {\textbf{q}'\rightarrow \textbf{q}}\limits ^{\scriptstyle t'\rightarrow t}}} (-i) \frac{i{\partial _t}+\omega _\textbf{q}}{2\omega _\textbf{q}} \frac{i{\partial _t}'-\omega _{\textbf{q}'}}{2\omega _{\textbf{q}'}} D^<_{\textbf{q}\textbf{q}'}(t,t') . \end{aligned}$$

The derivation of $D_{\textbf{q}\textbf{q}'}^<(t,t')$ is performed using standard methods and we consider the influence of the QD dynamics on the phonons. Thus, we perform an analysis based on the simplest approximation of $D_{\textbf{q}\textbf{q}'}^<$, given by

7a $$\begin{aligned} D^<_{\textbf{q}\textbf{q}'}(t,t')=&\delta _{\textbf{q}\textbf{q}'}d_\textbf{q}^<(t,t') + \int d^r_\textbf{q}(t,\tau )\Pi ^<_{\textbf{q}\textbf{q}'}(\tau ,\tau ')d^a_{\textbf{q}'}(\tau ',t') d\tau d\tau ' , \end{aligned}$$

7b $$\begin{aligned} \Pi _{\textbf{p}\textbf{q}}^<(t,t')=&(-i)\delta _{\textbf{p}\textbf{q}} \textrm{sp} \tilde{\varvec{\Lambda }}_\textbf{q}(t)\textbf{g}^<(t,t')\tilde{\varvec{\Lambda }}^\dagger _\textbf{q}(t')\textbf{g}^>(t',t) . \end{aligned}$$

In the self-energy $\Pi ^<_{\textbf{q}\textbf{p}}$ (Eq. (7b)), $\textbf{g}^{</>}$ denotes the lesser and greater Green functions for the unperturbed electrons in the QD, and $\textrm{sp}$ is the trace over spin-1/2 space. With the phonon Green function expressed in the form given in Eq. (7), $\omega _1$ becomes explicit in this expression. We justify this simplification by noting that we are only interested in the possibility of transferring excitations between the QD and the phonon reservoir, making the exact solutions and treatment of the equations of subordinate significance. The decoherence length can be expected to be substantial for insulators and semi-conductors, see, e.g., Ref.^22^. Therefore, we can safely omit decoherence related to electron-phonon interactions.

It is instructive to calculate the occupation $\langle {n^{(0)}_\textbf{q}}\rangle (t)$ for the unperturbed phonons, which is provided by the first term on the right hand side of Eq. (7a). The calculation is straight forward: using $d_\textbf{q}^<(t,t')=(-i)[n_B(\omega _\textbf{q})e^{-i\omega _\textbf{q}(t-t')}-n_B(-\omega _\textbf{q})e^{i\omega _\textbf{q}(t-t')}]$, where $n_B(\omega )$ is the Bose-Einstein distribution function, we obtain

8 $$\begin{aligned} \langle {n^{(0)}_\textbf{q}}\rangle (t)=&\lim _{t'\rightarrow t} (-i) \frac{i{\partial _t}+\omega _\textbf{q}}{2\omega _\textbf{q}} \frac{i{\partial _t}'-\omega _{\textbf{q}'}}{2\omega _{\textbf{q}'}} d^<_\textbf{q}(t,t') \\=& -\lim _{t'\rightarrow t} \frac{2\omega _\textbf{q}}{2\omega _\textbf{q}}\cdot \frac{-2\omega _\textbf{q}}{2\omega _\textbf{q}} n_B(\omega _\textbf{q})e^{-i\omega _\textbf{q}(t-t')} = n_B(\omega _\textbf{q}) , \end{aligned}$$

which is to be expected for the unperturbed phonon occupation number.

The correction $\langle {\delta n_\textbf{q}}\rangle (t)$ to the average occupation number caused by the time-dependent magnetic field can, furthermore, be written as

9 $$\begin{aligned} \langle {\delta n_\textbf{q}}\rangle (t)=&\lim _{{\mathop {\textbf{q}'\rightarrow \textbf{q}}\limits ^{\scriptstyle t'\rightarrow t}}} (-i) \frac{i{\partial _t}+\omega _\textbf{q}}{2\omega _\textbf{q}} \frac{i{\partial _t}'-\omega _{\textbf{q}'}}{2\omega _{\textbf{q}'}} \int d^r_\textbf{q}(t,\tau )\Pi ^<_{\textbf{q}\textbf{q}'}(\tau ,\tau ')d^a_{\textbf{q}'}(\tau ',t') d\tau d\tau ' \\=& i \int _{-\infty }^t \Pi ^<_{\textbf{q}\textbf{q}}(\tau ,\tau ') e^{i\omega _\textbf{q}(\tau -\tau ')} d\tau d\tau ' , \end{aligned}$$

where we have used, for instance, the equality $(i{\partial _t}-\omega _\textbf{q})d_\textbf{q}^r(t,t')/2\omega _\textbf{q}=(-i)\theta (t-t')e^{-i\omega _\textbf{q}(t-t')}$.

Physically, the lesser and greater Green functions ($\textbf{g}^{</>}$) quantify the density of occupied and unoccupied states in the QD, respectively. Hence, the self-energy $\Pi ^<_{\textbf{p}\textbf{q}}$ explicitly describes the dynamical transfer between the occupied and unoccupied states in the QD, along with its associated absorption and emission of energy. While the QD absorbs energy provided by the time-varying magnetic field, the emission processes induce the corresponding energy exchange between the phonon ground state and some excited state, as in Fig. 1.

Since the driving field is applied only to the QD, and we are interested in the pumping of phonons by this field, for ease of calculation we assume that the coupling parameters can be factorized as $\tilde{\varvec{\Lambda }}_\textbf{q}(t)=\tilde{\varvec{\Lambda }}(t)e^{i\textbf{q}\cdot \textbf{r}}$, with $\tilde{\varvec{\Lambda }}(t)=\lambda _0\sigma ^0+{\varvec{\lambda }}_1(t)\cdot {\varvec{\sigma }}$ (c.f., Eq. (3)). Hence, the self-energy pertaining to $B_1$ reduces to

10 $$\begin{aligned} \Pi ^<(t,t')=&\lambda _0^2j_0(t,t') + \lambda _0{\varvec{\lambda }}_1(t)\cdot \textbf{d}_L(t,t') + \lambda _0{\varvec{\lambda }}^\dagger _1(t')\cdot \textbf{d}_R(t,t') \nonumber \\ &+ J(t,t') {\varvec{\lambda }}_1(t) \cdot {\varvec{\lambda }}^\dagger _1(t') + \textbf{D}(t,t') \cdot \Bigl ( {\varvec{\lambda }}_1(t) \times {\varvec{\lambda }}^\dagger _1(t') \Bigr ) \nonumber \\ &+ {\varvec{\lambda }}_1(t) \cdot {\mathbb {I}}(t,t') \cdot {\varvec{\lambda }}^\dagger _1(t') . \end{aligned}$$

In these expressions, the functions $j_0$, $\textbf{d}_{L/R}$, *J*, $\textbf{D}$, and ${\mathbb {I}}$ are analogous to the electronically mediated indirect exchange interactions that are found between nuclear displacement and localized spin moments, see Ref.^19^. Despite the apparent similarity with the indirect exchange interaction between spin moments, here it is rather an electronically mediated connection between the electron-phonon couplings $\lambda _0$ and ${\varvec{\lambda }}_1(t)$ which, ultimately, provides an interaction between the phonon variables. In the next section, we go through the physical content of these interactions.

**Fig. 2:**
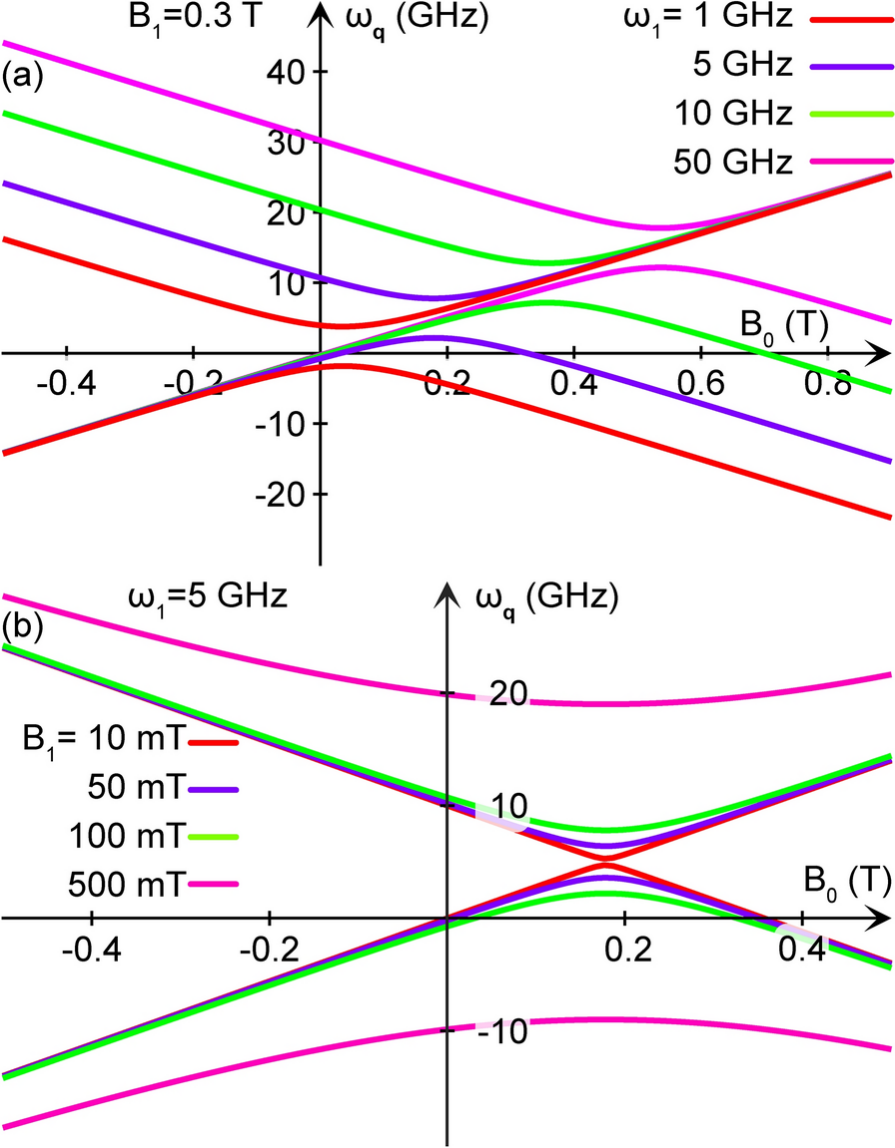
Plots of $\omega _\textbf{q}$, obtained from Eq. (13), as function of the static magnetic field $B_0$ for (**a**) $\omega _1\in \{1,\ 5,\ 10,\ 50\}$ GHz at $B_1=0.3$ T, and (**b**) $B_1\in \{10,\ 50,\ 100,\ 500\}$ mT at $\omega _1=5$ GHz. Here, we have also used the gyromagnetic ratio $g=2$.

Here, we concentrate on the impact of the processes involving both the electronic offset energy $\varepsilon _{+}-\varepsilon _{-}$ and the external frequency $\omega _1$. Such processes are mediated through the interactions *J*, $\textbf{D}$, and ${\mathbb {I}}$. Specifically, considering time-dependent processes imposed by the isotropic connection *J*, we have ${\varvec{\lambda }}_1(t)\cdot {\varvec{\lambda }}^\dagger _1(t')=\lambda _\perp ^2\cos \omega _1(t-t')$, $\lambda _\perp =|(\lambda _1^{(x)},\lambda _1^{(y)},0)|$. Hence, despite the influence of the external magnetic field, the self-energy is local in time, which allows the Fourier transform of $\Pi ^<_J(t,t')$ to be written as

11 $$\begin{aligned} \Pi _J^<(\omega )=&(-i) \pi \lambda _\perp ^2 \sum _{ss'=\pm 1} f(\varepsilon _{s})f(-\varepsilon _{{\bar{s}}}) \delta (\omega -2s|\tilde{\varvec{\varepsilon }}|+s'\omega _1) . \end{aligned}$$

This expression is obtained by noting that the bare QD propagator $\textbf{g}^{</>}(t,t')=(\pm i)\sum _{s=\pm 1}f(\pm \varepsilon _{s})(\sigma ^0+s{\hat{{\varvec{\varepsilon }}}}\cdot {\varvec{\sigma }})e^{-i\varepsilon _{s}(t-t')}/2$, where $\varepsilon _{s}=\varepsilon _{0}+s|\tilde{\varvec{\varepsilon }}|$, $\tilde{\varvec{\varepsilon }}=(g\mu _BB_1,0,g\mu _BB_0-\omega _1)/2$, $\hat{{\varvec{\varepsilon }}}=\tilde{\varvec{\varepsilon }}/|\tilde{\varvec{\varepsilon }}|$, and $f(\omega )$ is the Fermi-Dirac distribution function. Inserting this form for the self-energy into Eq. (9), the correction $\langle {\delta n_\textbf{q}}\rangle _J$ to the average occupation number caused by the isotropic connection becomes

12 $$\begin{aligned} \langle {\delta n_\textbf{q}}\rangle _J=&\frac{\lambda _\perp ^2}{2} \sum _{ss'} \frac{f(\varepsilon _{s})f(-\varepsilon _{{\bar{s}}})}{(\omega _1-s'[2s|\tilde{\varvec{\varepsilon }}|-\omega _\textbf{q}])^2+1/4\tau _\textbf{q}^2} , \end{aligned}$$

where the phonon life-time $\tau _\textbf{q}$ has been added to make the result regular. Note that the average occupation is strongly resonant at the driving frequencies $\omega _1=\pm (2|\tilde{{\varvec{\varepsilon }}}|-\omega _\textbf{q})$ and $\omega _1=\pm (2|\tilde{{\varvec{\varepsilon }}}|+\omega _\textbf{q})$.

The form of $\langle {\delta n_\textbf{q}}\rangle _J$ given in Eq. (12) clearly shows that it should be possible to tune the frequency $\omega _1$ to match one of the resonance conditions, thereby exciting a large number of phonons at energy $\omega _\textbf{q}$. The matching conditions are fulfilled for frequencies $\omega _1$ such that $\omega _1+s'(\omega _\textbf{q}\pm 2|\tilde{\varvec{\varepsilon }}|)=0$. This leads to the prediction that phonon modes should be excited at the energies

13 $$\begin{aligned} \omega _\textbf{q}=&\left| \omega _1\mp g\mu _B\sqrt{B_1^2+\left( B_0-\frac{\omega _1}{g\mu _B}\right) ^2} \right| . \end{aligned}$$

The induced phonon occupation has an upper limit, that is, $\langle {\delta n_\textbf{q}}\rangle _J\le 4\tau _\textbf{q}^2\lambda _\perp ^2$.

**Fig. 3:**
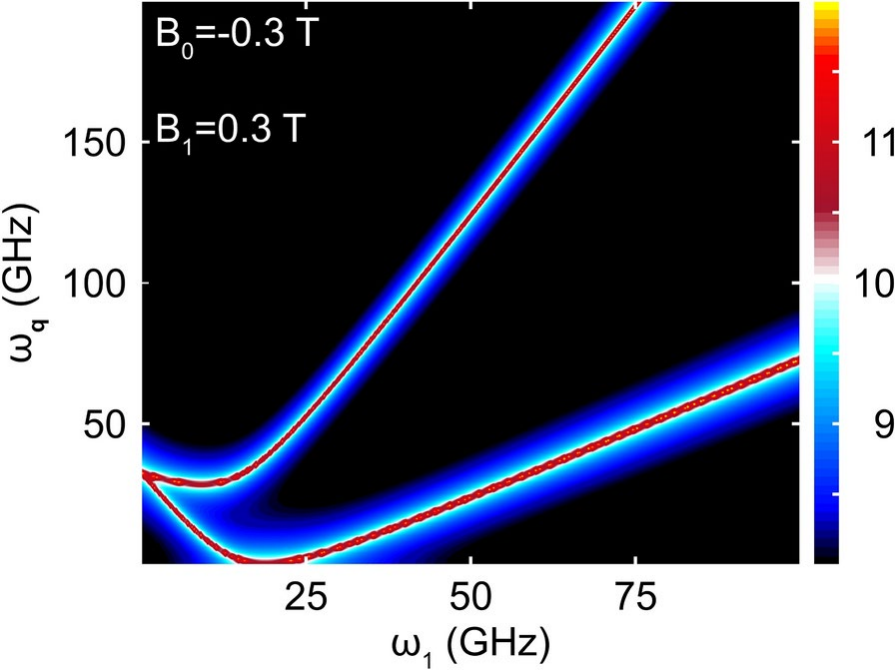
Acoustic phonon occupation number $\langle {n_\textbf{q}}\rangle _J$ (logarithmic scale) and its dependence on the frequency $\omega _1$ of the oscillating magnetic field (horizontal axis) and phonon frequency $\omega _\textbf{q}$ (vertical axis). Here, $B_0=-0.3$ T and $B_1=0.3$ T, whereas other parameters are $\varepsilon _{0}-\mu =0$, $\mu =0$, and the temperature $T=3$ K.

The plots in Fig. 2 show the generated phonon frequencies $\omega _\textbf{q}$, for various static and time-varying magnetic fields (see the caption for details). The separate branches associated with these two phonons approach one another at $\omega _1-g\mu _BB_0=0$, where their energy separation reaches a minimum value of $g\mu _BB_1$. The plots in Fig. 2a indicate how the resonances shift to larger field strengths $B_0$ with increasing $\omega _1$. In Fig. 2b, we highlight how the gap between the two phonon modes increases with increasing $B_1$.

In Fig. 3, the average phonon population $\langle {n_\textbf{q}}\rangle _J$ is plotted as function of $\omega _1$ and $\omega _\textbf{q}$ (see the figure caption for further details). The two excitation branches correspond to the $+$ (−) solution in Eq. (13) and can be identified with the upper (lower) branch in Fig. 3. For low pumping frequencies, $\omega _1\rightarrow 0$, the two phonon excitation lines coalesce at the energy $\omega _\textbf{q}=g\mu _BB_0$, as demonstrated near the bottom-left corner of Fig. 3.

### Electronically mediated exchange interactions

As mentioned above, the expressions for the functions $j_0$, $\textbf{d}_{L/R}$, *J*, $\textbf{D}$, and ${\mathbb {I}}$ are analogous to the electronically mediated indirect exchange interactions that are found between nuclear displacement and localized spin moments, see Ref.^19^. Here, however, the quantities that interact cannot be regarded as spins in the conventional sense. Rather, there is an electronically mediated connection between the electron-phonon coupling parameters ($\lambda _0$ and ${\varvec{\lambda }}_1$). Nevertheless, adopting the theory developed in Ref.^19^ and viewing the parameters $\lambda _0$ and ${\varvec{\lambda }}_1$ as representing the nuclear-displacement and localized-spin degrees of freedom, respectively, lends itself to interpreting the interactions in a physically viable sense.

Before going into the discussion of the interactions and their corresponding properties, we make use of the fact that the Green function $\textbf{g}$ can be expanded in terms of its charge, $g_0$, and spin, $\textbf{g}_1$, components, such that $\textbf{g}=g_0\sigma ^0+\textbf{g}_1\cdot {\varvec{\sigma }}$. These functions expressed in terms of the bare propagator $\textbf{g}^{</>}(t,t')=\textbf{g}^{</>}(t-t')$, which is local in time.

First, the interaction $j_0(t,t')$ constitutes a connection between a pair of the scalar electron-phonon coupling parameter $\lambda _0$, and is given by $j_0(t,t')=(-i)2[g^<_0(t,t')g^>_0(t',t)+\textbf{g}^<_1(t,t')\cdot \textbf{g}^>_1(t',t)]$. The form of this connections is similar to the electronic contribution to the interatomic force constant^19^. In terms of the propagator $\textbf{g}$, it is easily verified that $j_0$ is time-independent. Hence, there is no pumping mechanism associated with this term.

Second, the processes coupled to $\textbf{d}_\chi$, $\chi =L,R$, open up the possibility of directly pumping phonons of frequency $\omega _1$. However, since these phonons are at the same frequency as the driving signal, there is no ESR arising from this contribution, which may be seen from, e.g.,

14 $$\begin{aligned}&\lambda _0{\varvec{\lambda }}_1(t)\cdot \textbf{d}_L(t,t')= (-i)2 \lambda _0{\varvec{\lambda }}_1(t)\cdot \Bigl ( \textbf{g}^<_1(t,t')g^>_0(t',t) + g^<_0(t,t')\textbf{g}^>_1(t',t) + i\textbf{g}^<_1(t,t')\times \textbf{g}^>_1(t',t) \Bigr ) \nonumber \\=&(-i)2 \lambda _0{\varvec{\lambda }}_1(t)\cdot \int \Bigl ( \textbf{g}^<_1(\omega )g^>_0(\omega ') + g^<_0(\omega )\textbf{g}^>_1(\omega ') + i\textbf{g}^<_1(\omega )\times \textbf{g}^>_1(\omega ') \Bigr ) e^{-i(\omega -\omega ')(t-t')} \frac{d\omega }{2\pi } \frac{d\omega '}{2\pi } . \end{aligned}$$

Hence, the electronically mediated connection between the couplings $\lambda _0$ and ${\varvec{\lambda }}_1(t)$ has the trivial time-dependence of the vector coupling ${\varvec{\lambda }}_1(t)$ itself. The properties of the interaction, $\textbf{d}_\chi$, hence, resemble the properties that are already provided by the electronically mediated spin-lattice coupling, and do not contribute any additional physics.

Finally, the connections between a pair of the vector couplings ${\varvec{\lambda }}_1(t)$ and ${\varvec{\lambda }}_1(t')$ are given by

15a $$\begin{aligned} J(t,t')=&(-i)2\Bigl ( g^<_0(t,t')g^>_0(t',t)-\textbf{g}^<_1(t,t')\cdot \textbf{g}^>_1(t',t) \Bigr ) , \end{aligned}$$

15b $$\begin{aligned} \textbf{D}(t,t')=&2\Bigl ( g^<_0(t,t')\textbf{g}^>_1(t',t)-\textbf{g}^<_1(t,t')g^>_0(t',t) \Bigr ) , \end{aligned}$$

15c $$\begin{aligned} {\mathbb {I}}(t,t')=&(-i)2\Bigl ( \textbf{g}^<_1(t,t')\textbf{g}^>_1(t',t)+\textbf{g}^<_1(t',t)\textbf{g}^>_1(t,t') \Bigr ) . \end{aligned}$$

These three connections are providing, isotropic (*J*), anti-symmetric anisotropic ($\textbf{D}$), and symmetric anisotropic (${\mathbb {I}}$) interactions between ${\varvec{\lambda }}_1(t)$ and ${\varvec{\lambda }}_1(t')$. In this sense, these connections are reminiscent of the Heisenberg, Dzyaloshinskii-Moriya, and Ising interactions between spin moments. The difference in the present context is that the connections do not constitute interactions between spin variables. 

The isotropic connection is given by

16 $$\begin{aligned}&J(t,t'){\varvec{\lambda }}_1(t)\cdot {\varvec{\lambda }}^\dagger (t')= (-i)2\lambda _\perp ^2 \int \Bigl ( g^<_0(\omega )g^>_0(\omega ')-\textbf{g}^<_1(\omega )\cdot \textbf{g}^>_1(\omega ') \Bigr ) e^{-i(\omega -\omega ')(t-t')} \cos \omega _1(t-t') \frac{d\omega }{2\pi } \frac{d\omega '}{2\pi } \nonumber \\=&(-i)\lambda _\perp ^2 \int \Bigl ( g^<_0(\omega )g^>_0(\omega ')-\textbf{g}^<_1(\omega )\cdot \textbf{g}^>_1(\omega ') \Bigr ) \Bigl ( e^{-i(\omega -\omega '-\omega _1)(t-t')} + e^{-i(\omega -\omega '+\omega _1)(t-t')} \Bigr ) \frac{d\omega }{2\pi } \frac{d\omega '}{2\pi } , \end{aligned}$$

which is translationally invariant in time. Nevertheless, the harmonic driving frequency $\omega _1$ is mixed with the electronic energies such that the system can be driven into resonance. Notice that here we have made use of the form of $\tilde{\varvec{\Lambda }}_\textbf{q}(t)$ given in Eq. (3).

The asymmetric anisotropic contribution is given by

17 $$\begin{aligned} \textbf{D}(t,t')\cdot \Bigl ({\varvec{\lambda }}_1(t)\times {\varvec{\lambda }}_1^\dagger (t')\Bigr )=&2 \int \Bigl ( g_0^<(\omega )\textbf{g}_1^>(\omega ') - \textbf{g}_1^<(\omega )g_0^>(\omega ') \Bigr ) e^{-i(\omega -\omega ')(t-t')} \cdot \Bigl ( {\varvec{\lambda }}_1(t)\times {\varvec{\lambda }}_1(t') \Bigr ) \frac{d\omega }{2\pi } \frac{d\omega '}{2\pi } . \end{aligned}$$

This contribution is not translationally invariant with time, unless the vector product vanishes. This can be understood by again using ${\varvec{\lambda }}_1(t)$ as given in the form in Eq. (3) ($\lambda ^{(z)}_\textbf{q}=0$). Then, the vector product ${\varvec{\lambda }}_1(t)\times {\varvec{\lambda }}_1^\dagger (t')=(-i)\hat{\textbf{z}}\lambda _\perp ^2\sin \omega _1(t+t')$ and the spin-dependent part $\textbf{g}_1$ of the electron Green function can be factorized on the form $\textbf{g}_1=g_1{\hat{{\varvec{\varepsilon }}}}$. The time-dependence of this asymmetric anisotropic contributions is then given by

18 $$\begin{aligned} \textbf{D}(t,t')\cdot \Bigl ({\varvec{\lambda }}_1(t)\times {\varvec{\lambda }}_1^\dagger (t')\Bigr )=&\lambda _\perp ^2 \hat{\textbf{z}}\cdot {\hat{{\varvec{\varepsilon }}}} \int \Bigl ( g_1^<(\omega )g_0^>(\omega ') - g_0^<(\omega )g_1^>(\omega ') \Bigr )\\&\times \Bigl ( e^{-i(\omega -\omega '-\omega _1)(t-t')+i2\omega _1t'} - e^{-i(\omega -\omega '+\omega _1)(t-t')-i2\omega _1t'} \Bigr ) \frac{d\omega }{2\pi } \frac{d\omega '}{2\pi } . \end{aligned}$$

This contribution has an additional time-dependence that prevents us from Fourier transforming it in similar fashion to what was done for the isotropic contribution. However, by neglecting factors with the frequency $2\omega _1$, and thereby resorting to the rotating-wave approximation, the asymmetric anisotropic interaction reduces to a form that provides the same type of time-dependence as the isotropic term. Consequently, it does not introduce any new time, or, energy scale.

Finally, the symmetric anisotropic contribution is of the form

19 $$\begin{aligned} {\varvec{\lambda }}_1(t)\cdot {\mathbb {I}}(t,t')\cdot {\varvec{\lambda }}_1^\dagger (t')=&(-i)4 \int g_1^<(\omega )g_1^>(\omega ) {\varvec{\lambda }}_1(t)\cdot {\hat{{\varvec{\varepsilon }}}} {\hat{{\varvec{\varepsilon }}}}\cdot {\varvec{\lambda }}_1^\dagger (t') e^{-i(\omega -\omega ')(t-t')} \frac{d\omega }{2\pi } \frac{d\omega '}{2\pi } . \end{aligned}$$

Expanding the scalar products here, it is straight forward to see that the full time-dependence of the symmetric contribution is given by the expression

20 $$\begin{aligned}&{\varvec{\lambda }}_1(t)\cdot {\mathbb {I}}(t,t')\cdot {\varvec{\lambda }}_1^\dagger (t')= (-i)4\lambda _\perp ^2 \int g_1^<(\omega )g_1^>(\omega ) e^{-i(\omega -\omega ')(t-t')} \nonumber \\ &\times \biggl ( \cos \Bigl (\omega _1(t+t')+2\varphi \Bigr ) + i\sin \Bigl (\omega _1(t+t')+2\varphi \Bigr )\cos 2\phi + \cos \omega _1(t-t')\sin 2\phi \biggr ) \sin ^2\theta \frac{d\omega }{2\pi } \frac{d\omega '}{2\pi } , \end{aligned}$$

where $\tan \varphi =\lambda _1^{(y)}/\lambda _1^{(x)}$ and ${\hat{{\varvec{\varepsilon }}}}=(\cos \phi \sin \theta ,\sin \phi \sin \theta ,\cos \theta )$. It is clear from this expression that the time-dependence of the symmetric anisotropic contribution is of the same form of that which appears in the other interactions.

### Phonon flux

Next, we determine the phononic flux $\textbf{j}_Q(x)$, $x=(\textbf{r},t)$, injected into a phonon reservoir by a local source of phonons. The solution to this problem is of interest as it provides an estimate of how the phonons generated in the ESR process spatially decay as they propagate away from the QD. To solve this problem, we start from the generic form for a particle current, given by

21 $$\begin{aligned} \textbf{j}_Q(x)\sim&\lim _{\textbf{r}'\rightarrow \textbf{r}} \lim _{t'\rightarrow t} \Bigl ( \nabla _\textbf{r}- \nabla _{\textbf{r}'} \Bigr ) {\mathcal {G}}^<(x,x') . \end{aligned}$$

Here, ${\mathcal {G}}^<(x,x')=\sum _{qq'}{\mathcal {G}}_{qq'}^<(t,t')e^{i\textbf{q}\cdot \textbf{r}-i\textbf{q}'\cdot \textbf{r}'}/{\mathcal {N}}$, where ${\mathcal {N}}$ is the number of phonons in reciprocal space, defines the occupation of the phonon distribution in coordinate space in terms of the correlation function ${\mathcal {G}}_{qq'}^<(t,t')=(-i)\langle {b_{q'}^\dagger (t')b_q(t)}\rangle$. In this representation, $p=(\textbf{q},\mu )$ where $\mu$ is the phonon branch index is included for completeness. With these definitions we express the phonon current as

22 $$\begin{aligned} \textbf{j}_Q(x)=&\frac{i}{{\mathcal {N}}} \sum _{qq'} \Bigl ( \textbf{q}+ \textbf{q}' \Bigr ) {\mathcal {G}}_{qq'}^<(t,t) e^{i(\textbf{q}-\textbf{q}')\cdot \textbf{r}} , \end{aligned}$$

where it, in analogy with Eq. (6), can also be seen that

23 $$\begin{aligned} {\mathcal {G}}_{qq'}^<(t,t')=&- \frac{1}{4\omega _q\omega _{q'}} \Bigl ( i{\partial _t}+\omega _q \Bigr ) \Bigl ( i\partial _{t'}-\omega _{q'} \Bigr ) D^<_{qq'}(t,t') . \end{aligned}$$

This last equality provides the connection between the correlation function ${\mathcal {G}}_{qq'}^<$ and the lesser phonon Green function $D_{qq'}^<$ discussed above. Incorporating our results from the discussion of the phonon excitations generated through processes captured in the self-energy $\Pi _J$, the phonon flux is reduced to to the time-independent expression

$$\begin{aligned} \textbf{j}_Q(\textbf{R})=&- \frac{\pi \lambda _\perp ^2}{{\mathcal {N}}^2} \textrm{Im}\sum _{\textbf{q}\textbf{q}'} \sum _{ss'=\pm 1} \textbf{q}e^{i\textbf{q}\cdot (\textbf{r}-\textbf{r}_0)-i\textbf{q}'\cdot (\textbf{r}-\textbf{r}_0)} \frac{f(\varepsilon _{s})f(-\varepsilon _{{\bar{s}}})}{(2s|\tilde{\varvec{\varepsilon }}|+s'\omega _1+\omega _q-i\delta )(2s|\tilde{\varvec{\varepsilon }}|+s'\omega _1+\omega _{q'}+i\delta )} , \end{aligned}$$

where $\delta >0$ infinitesimal. Restricting our analysis to the case of two-dimensional acoustic phonons, such that $\omega _q=\gamma q$, the current can finally be expressed in the form

24 $$\begin{aligned} \textbf{j}_Q(\textbf{R})=&\frac{\pi \lambda _\perp ^2}{8} \sum _{ss'=\pm 1} \omega _{ss'}^3 f(\varepsilon _{s})f(-\varepsilon _{{\bar{s}}}) \Bigl ( J_0(\omega _{ss'}R_0)J_1(\omega _{ss'}R_0) + Y_0(\omega _{ss'}R_0)Y_1(\omega _{ss'}R_0) \Bigr ) {\hat{\textbf{R}}}_0 , \end{aligned}$$

where $\textbf{R}_0=\textbf{r}-\textbf{r}_0$, ${\hat{\textbf{R}}}_0=\textbf{R}_0/R_0$, $R_0=|\textbf{R}_0|$, $\omega _{ss'}=(2s|\tilde{\varvec{\varepsilon }}|+s'\omega _1)/\gamma$, and $J_m$ and $Y_m$ ($m=0,1$) are the Bessel and Neumann functions, respectively. Equation (24) shows that there is a phonon flux emitted concentrically from the QD, with energy $\omega _{ss'}=2s|\tilde{\varvec{\varepsilon }}|+s'\omega _1$. In other words, due to the transition from the higher Zeeman state to the lower one, the emitted energy quantum $\omega _{ss'}$ is absorbed by the phonon distribution, thereby generating a flux that transports the excess energy into the phonon reservoir. The asymptotic distribution of the phonon flux for large $R_0$ can be written as

25 $$\begin{aligned} \textbf{j}_Q(x)\approx&\frac{1}{2} \biggl ( \frac{\lambda _\perp }{2R_0} \biggr )^2 \sum _{ss'=\pm 1} \frac{f(\varepsilon _{s})f(-\varepsilon _{{\bar{s}}})}{\omega _{ss'}} {\hat{\textbf{R}}}_0 . \end{aligned}$$

This non-oscillatory decay is expected for bosonic-type propagation, in contrast to the similary quadratic but oscillatory decay associated with fermionic propagation (see for instance, Ref.^23^).

The scheme that we have proposed is one that is highly amenable to implementation for on-chip phonon sourcing. Quantum-dot technology has advanced considerably over the past few decades and the application of these structures to solid-state ESR is well established. Implementations of the phonon source can be envisaged based on either split-gate technology, or self-assembled QDs, while enhancing the required Zeeman splitting by appropriate choice of (large g-factor) materials. With regards to the model parameters utilized in the calculations of Figs. 2 and 3, the static magnetic field required to realize the avoided crossing in Fig. 2b, should be readily accessible using a permanent nanomagnet integrated with the quantum dot. Furthermore, since the calculations of Fig. 3 show well resolved phonon resonances for a bath temperature of 3 K, the need for challenging implementation at milli-Kelvin temperatures can be avoided. Single-phonon sourcing should also be possible, by subjecting the system to a suitably designed magnetic field pulse, rather than the harmonic (continuous) signal considered here. Finally, we note that while our calculations reveal the presence of phonon excitation at *two* distinct frequencies, one of these modes may be suppressed by suitable materials choice or by nanostructured patterning. In the former case, the objective would be to select a host material whose phononic density of states vanishes in the energy range associated with one of the two modes. In the latter case, one would implement a similar *phononic gap* by periodically patterning the crystal, in direct analogy with the case of photonic crystals.

## Conclusions

In summary, we have discussed the possibility of realizing an electrically driven source of phonons, implemented with a QD whose Zeeman-split electronic levels are coupled to a phonon reservoir. Application of a time-varying, transverse, magnetic field couples these levels, with the frequency of this field serving as the control parameter that allows ESR to be achieved. The coupling of the electron levels in the QD to the phonons of the host material then provides a means to target and excite phonons with specific energy, which can be sourced into the phonon reservoir.

## Data Availability

All data generated or analysed during this study are included in this published article.
